# Dentists Make Larger Holes in Teeth Than They Need to If the Teeth Present a Visual Illusion of Size

**DOI:** 10.1371/journal.pone.0077343

**Published:** 2013-10-23

**Authors:** Robert P. O’Shea, Nicholas P. Chandler, Rajneesh Roy

**Affiliations:** 1 Department of Psychology, University of Otago, Dunedin, New Zealand; 2 Discipline of Psychology and Cognitive Neuroscience Research Cluster, School of Health and Human Sciences, Southern Cross University, Coffs Harbour, Australia; 3 Sir John Walsh Research Institute, Faculty of Dentistry, University of Otago, Dunedin, New Zealand; University of Leuven, Belgium

## Abstract

**Background:**

Health care depends, in part, on the ability of a practitioner to see signs of disease and to see how to treat it. Visual illusions, therefore, could affect health care. Yet there is very little prospective evidence that illusions can influence treatment. We sought such evidence.

**Methods and Results:**

We simulated treatment using dentistry as a model system. We supplied eight, practicing, specialist dentists, endodontists, with at least 21 isolated teeth each, randomly sampled from a much larger sample of teeth they were likely to encounter. Teeth contained holes and we asked the endodontists to cut cavities in preparation for filling. Each tooth presented a more or less potent version of a visual illusion of size, the Delboeuf illusion, that made the holes appear smaller than they were. Endodontists and the persons measuring the cavities were blind to the parameters of the illusion. We found that the size of cavity endodontists made was linearly related to the potency of the Delboeuf illusion (*p*<.01) with an effect size (Cohen’s *d)* of 1.41. When the illusion made the holes appear smaller, the endodontists made cavities larger than needed.

**Conclusions:**

The visual context in which treatment takes place can influence the treatment. Undesirable effects of visual illusions could be counteracted by a health practitioner’s being aware of them and by using measurement.

## Introduction

**Figure 1:**
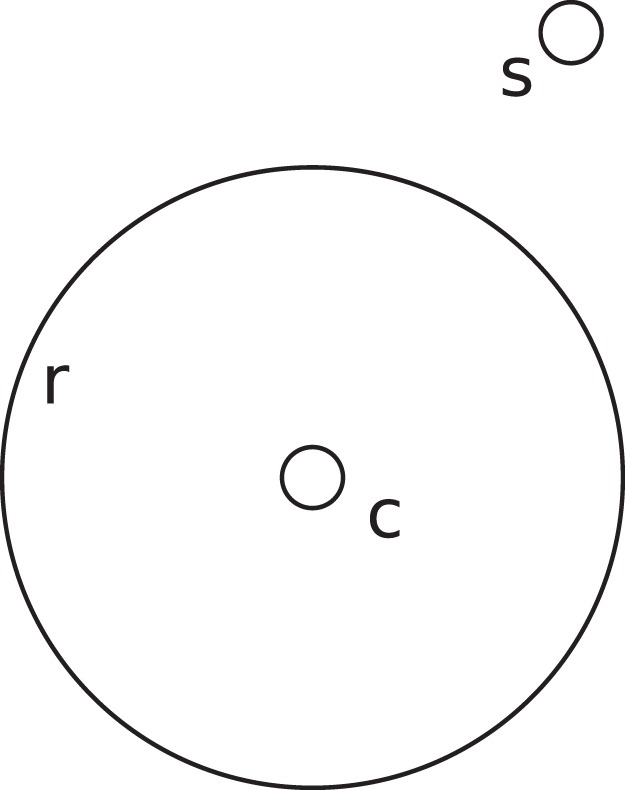
A version of the Delboeuf illusion. Circles *c* and *s* are the same size, but *c* appears smaller when surrounded by a larger circle, *r*. If one adjusts the size of *c* to appear the same as that of *s*, one makes it larger than it should be.

Does visual perception [1], [2] affect treatment by health-care providers? In particular, do visual illusions (also known as optical illusions and as geometrical illusions) affect treatment? In visual illusions, what one perceives is different from what is in front of one’s eyes [3], [4], [5], [6]. For example, surrounding a small, *test* circle by a larger one, called the *inducer,* can make the test circle appear smaller (or larger) than it is–the Delboeuf illusion (Figure 1) [7], [8], [9]. Because most health practitioners use their eyes to look for signs of disease and to guide treatment [10], visual illusions have the potential to affect health care.

### Properties of the Delboeuf Illusion

**Figure 2:**
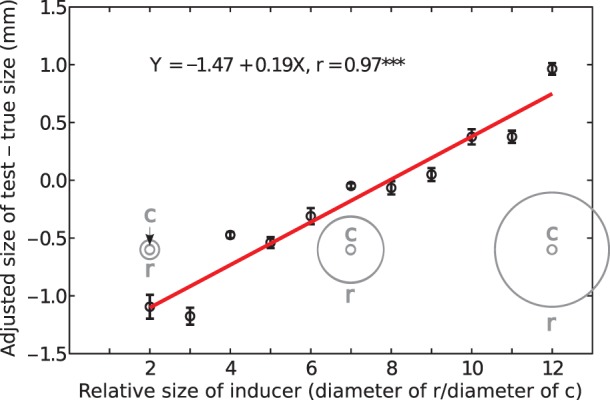
Data from one of the participants in our study to conventional displays of the Delboeuf illusion. The inner circle (*c*) had a fixed diameter of 10 mm. The outer, inducing circle had a range of values from 20 mm (relative inducer size of 2) to 120 mm (relative inducer size of 12) in 10-mm steps. The participant adjusted the size of a circle (*s*; not shown) in the lower left of a computer-monitor screen to match the apparent size of *c*. Three such stimuli are illustrated, spatially to scale, in grey–one in which the relative inducer size is 2, one in which the relative inducer size is 7, and one in which the relative inducer size is 12. The small plot circles are the mean differences between the adjusted size of *s* and its true size (10 mm) for each value of relative inducer size from three trials each (the participant received the trials in a completely random order); the vertical bars are standard errors. The red line is the statistically significant, best-fitting, positively sloping, linear function.

The Delboeuf illusion is one example of visual illusions in which the context of an object affects its perceived size. When the context is large, the object appears smaller than it is [3], [11], [12]. These illusions occur with any shapes and are most potent when the shapes are similar [13], [14], [15]. The Delboeuf illusion depends on the ratio of the inducer’s diameter (*r*) to that of the test’s (*c*) [11]. We call this ratio the *relative size of the inducer*. When the relative size of the inducer is 1.5, the test appears larger than it is (opposite to that visible in Figure 1). This is referred to as *assimilation*; it diminishes with increases of the relative size of the inducer to about 6.5, at which the test appears to have an accurate size. When the relative size of the inducer is larger than 7, the test appears increasingly smaller than it is (as demonstrated in Figure 1). This is referred to as *repulsion*. When the size of the test is adjusted to appear equal to a visible or remembered standard, its diameter has a positively sloping linear relationship to the relative size of the inducer (see Figure 2).

### Prior Research on Illusions in Health Care

For a health practitioner, underestimating the size of something, be it a vessel, a tumour, or other lesion, could have serious consequences. Indeed, visual illusions have been implicated in errors of diagnosis [16], [17], [18], [19], [20], [21] and of treatment [2], [22].

One strand of research implicating visual illusions in errors of diagnosis and of treatment has involved techniques such as reviewing cases in which errors occurred and deciding on the primary cause. Although such techniques have good external validity, they can be affected by various biases, such as hindsight bias, and are correlational. For a study to have good internal validity, it needs to use a technique in which some aspect of the information available to a practitioner is systematically manipulated to determine how this affects diagnosis or treatment. Yet one cannot use classic experimental design to research visual illusions as causes of diagnosis or treatment errors for ethical reasons. If one reasonably suspected that an illusion could cause an error, it would be unethical to expose practitioners and their patients to that illusion in a clinical trial. What is required is some sort of simulation.

### Simulation Studies in Health Care

Simulation of conditions for diagnosis has involved showing practitioners video displays of actors portraying patients; this has shown various non-medical influences, such as age of the health-care provider and that of the patient, on diagnosis and decisions [23], [24], [25]. Although studies have suggested that visual illusions could affect diagnosis [16], [17], [18], [19], [20], [21], we are unaware of any that use simulation to take a prospective approach.

Simulation of conditions for treatment has been used to train practitioners [26], [27], [28] and to test the influence of human factors on treatment [29]. But again, as far as we are aware, this approach has never been used to test the influence of visual illusions. We tried to achieve good internal and external validity by using dentistry as our model of treatment. We were interested in determining whether the Delboeuf illusion could cause dentists to make larger cavities than they should.

### Dentistry as a Model of Treatment (and a Tutorial on Apicectomy)

A very similar display to that shown in Figure 1 is seen by specialist dentists, endodontists, during the most common endodontic surgical procedure: apicectomy [30].

**Figure 3:**
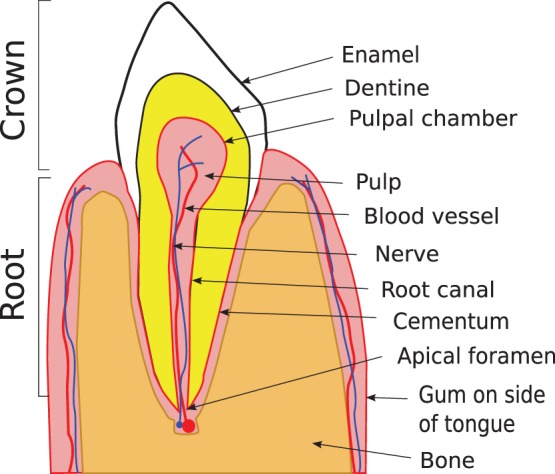
Schematic representation of a cross-section of a single-rooted tooth, such as a canine. The gum on the left is on the side of the lips; the gum on the right is on the side of the tongue.

The tooth consists of a *crown*–the part of the tooth that is visible in the mouth–and the *root* or *roots*. In Figure 3, we have shown what a single-rooted tooth (as used in our study), such as a canine, would look like if sectioned through the crown and root, perpendicular to the axis of the jaw at that point. The crown consists of external *enamel* over *dentine*. The dentine is produced and nourished by the *pulp* of the tooth; in the crown it is enclosed within the *pulpal chamber.*

The root consists of dentine over pulp; the pulp is confined within the *root canal*. The dentine is coated with *cementum*. The root anchors the tooth into the bone of the jaw and allows blood vessels and nerve fibres to connect, at the *apical foramen*, the pulp with the body’s circulatory and nervous systems respectively. [31].

**Figure 4:**
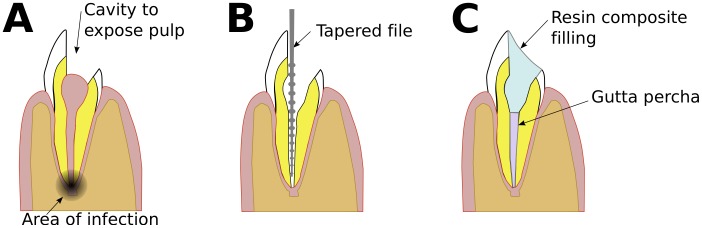
Schematic representation of the same tooth from Figure 3, illustrating the three main stages in root-canal therapy. A. The tooth with infection in the apical part of the root following death of the pulp. The dentist begins by making a cavity in the crown, entering the pulp space. B. The dentist cleans the root canal, and some of the surface of the canal, using fine, tapered files. The dentist also treats the infection with medication, leaving it there for one week. C. The dentist fills the canal with gutta percha and the crown with a composite resin.

Sometimes, the apical part of a tooth can become infected, usually from decay in the crown or from trauma to the tooth. Infection can kill the pulp and lead to an abscess or cyst at the apex of the root (Figure 4A). The first treatment is via the crown, so called root-canal treatment (Figure 4B). The dentist makes a *cavity* in the crown and clears out the pulpal chamber. Then he or she uses tiny, tapered files to clean the root canal as well as taking off some small amount of its interior surface. The dentist may also instil medication to treat any remaining infection, leaving it there for about one week before filling the canal, usually with gutta percha, and sealing the crown with a filling, usually of a resin composite (Figure 4C). Root-canal treatment has a success rate of over 95%, in which case the abscess resolves and the bone will heal.

**Figure 5:**
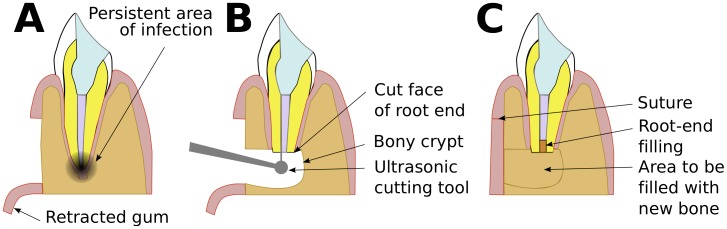
Schematic representation of the same tooth from Figure 4C, of the three main stages in apicectomy. A. The tooth with persistent infection in the apical part of the root. The specialist dentist, endodontist, begins by resecting (*reflecting*) the gum, exposing the bone of the lower jaw. B. The endodontist removes bone to expose the apical part of the root, creating the bony crypt. Then the endodontist removes and discards the apical 3 mm of the root, leaving the cut face. Then the endodontist uses a very fine, ultrasonic cutting tip to prepare a cavity in the root canal. C. The endodontist fills the cavity and sutures the gum. Eventually the surrounding bone will grow to fill the bony crypt.

In a few cases root-canal treatment fails, often due to microbes’ remaining in the complex canal morphology present in the terminal few millimeters of the root, so that disease in the bone around the tooth persists (Figure 5A). In the first instance, the root-canal treatment is repeated, but if this is unsuccessful or not technically feasible then the tooth has to be removed or an apicectomy performed [32], done by a specialist endodontist. The endodontist cuts a flap in the gum to expose the bone over the apex of the root, then drills out a small amount of the bone of the jaw to create a space around the apex of the root, the *bony crypt* (Figure 5B). Then he or she cuts off and discards 3 mm of the apex of the root. Cutting off the apex removes any infected material in it. As well, the endodontist, usually guided by what he or she can see via a dental micromirror, then uses an instrument with an ultrasonically-powered cutting tip to remove a small portion of the surface of the root canal, in preparation for a *root-end filling* that will seal the tooth. This filling is usually of mineral trioxide aggregate (MTA); it should be as small as possible to make an effective seal [31], [33]. Then the endodontist sutures the gum (Figure 5C). If the procedure is successful, the bony crypt is eventually filled by regenerated bone.

### How we Tested the Effects of the Delboeuf Illusion on Treatment

When an endodontist makes a cavity in the root canal (Figure 5B) he or she is effectively adjusting the size of *c* in Figure 1. The endodontist can also see all of the cut face of the root, making the Figure 1’s inducer, *r*, easily visible. If the relative size of *r* is suitable, the Delboeuf illusion might make *c* look smaller than it really is, prompting the endodontist to make the cavity larger than necessary.

We prepared teeth to look like what an endodontist sees when making a cavity in a root canal (see Methods). We achieved internal validity for our simulation by randomly varying the size of teeth and their canals. Endodontists and the persons measuring the cavities were blind to the parameters of the illusion. We achieved external validity by recruiting endodontists who perform this sort of operation routinely, by providing them with real human teeth randomly sampled from the population of teeth they would encounter, and by having them make the cavities with their usual instruments and viewing aids in their own surgeries and in their own time. If the Delboeuf illusion affects treatment, we can predict that the size of cavities the endodontists make should be positively sloping, linear functions of the relative size of the roots.

## Methods

### Ethics Statement

The study was performed in accordance with the ethical standards laid down in the Declaration of Helsinki [34]. Ethical approval was granted by the University of Otago Ethics Committee. We obtained written informed consent from each participant. We did not tell participants the aim of the experiment; we told them only of what was involved in participating.

### Participants

Participants were eight experienced endodontic specialists in private practice in New Zealand. This represents about 50% of all practising endodontists in the country in 2006 [35]. They had good eyesight with or without correction. Mean (SD) age was 43.50 (4.66) years; specialist experience was 13.25 (4.98) years. We tested three endodontists (A–C) in 2002 and five (1–5) in 2006.

### Teeth

The teeth were extracted, single-rooted teeth with normal apices, taken from a large pool of extracted teeth stored in normal saline. All had straight roots, closed apices, and no fractures or cracks (established by examining them with magnification and under optimum lighting). We root-filled and prepared the teeth (as shown in Figure 4) so they appeared to the endodontists as they would when they performed an apicectomy. We confirmed that each canal was open by passing a size-15 root-canal file (K-file, Dentsply Maillefer, Ballaigues, Switzerland) through the apical foramen until it was just visible. We then root-canal treated all the roots, adopting a working length 1 mm short of this length. We enlarged the canals with a series of root-canal files of increasing diameter to generate a continuous, funnel-shape for the entire root-canal space. At the apical termination of the canals we used a size 40 root canal file. We then filled the prepared canals with gutta percha and AH Plus sealer (Dentsply). We then stored the roots in a humid environment for 48 hours to ensure set of materials.

**Figure 6:**
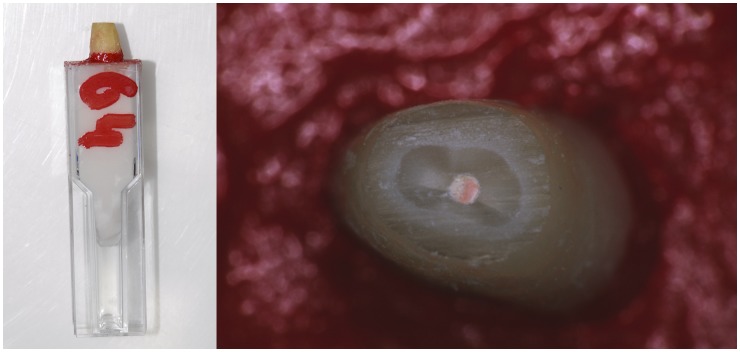
A prepared tooth, with a resected root end and a canal filled with gutta percha, mounted in a tube for work by an endodontist. On the left is the side view. On the right is a magnified plan view of the resected root end on its painted, red background of resin.

We examined the roots again for fractures, replacing any teeth if necessary. We embedded each tooth in an optical density tube using self-curing acrylic resin. Once set, we painted the resin surrounding the root-end red to provide contrast and to simulate the context of the tooth in the bony crypt. To simulate the apicectomy, we resected the apical 3 mm at a 90° angle to the root axis using a high-speed diamond bur with water irrigation. See Figure 6 for illustrations of a prepared root end.

**Figure 7:**
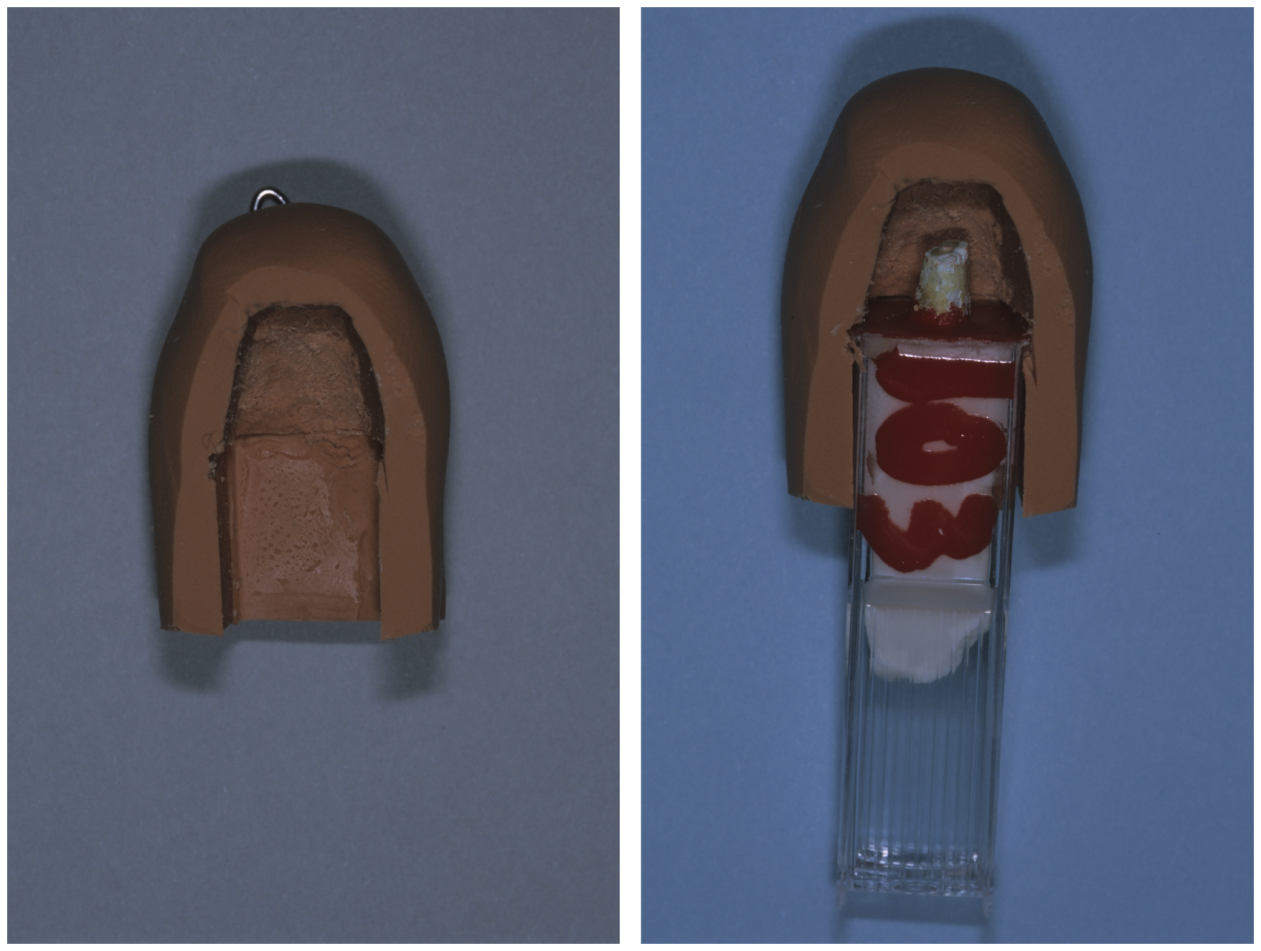
Illustration of the simulated bony crypt. Left: The simulated bony crypt. Right: The simulated bony crypt in place over a prepared resected root end.

For a further simulation of working within a bony crypt, five endodontists (1–5) worked on teeth in a housing made of silicone putty. The housing fitted around the root ends to limit access and visibility (Figure 7). The other three endodontists (A–C) worked without this. Use of the simulated bony crypt did not affect the results.

### Procedure

We supplied at least 21 teeth to each endodontist and asked him or her to prepare a cavity in the root end of each tooth ready to receive a filling. We emphasized that endodontists should cut conservative (minimal) cavities. Endodontists cut the cavities in their own surgeries in their own time; cavities would have taken no more than two minutes to prepare. Each endodontist worked as he or she would usually work on such teeth, using dental mirrors and any usual magnification.

### Measurement

**Figure 8:**
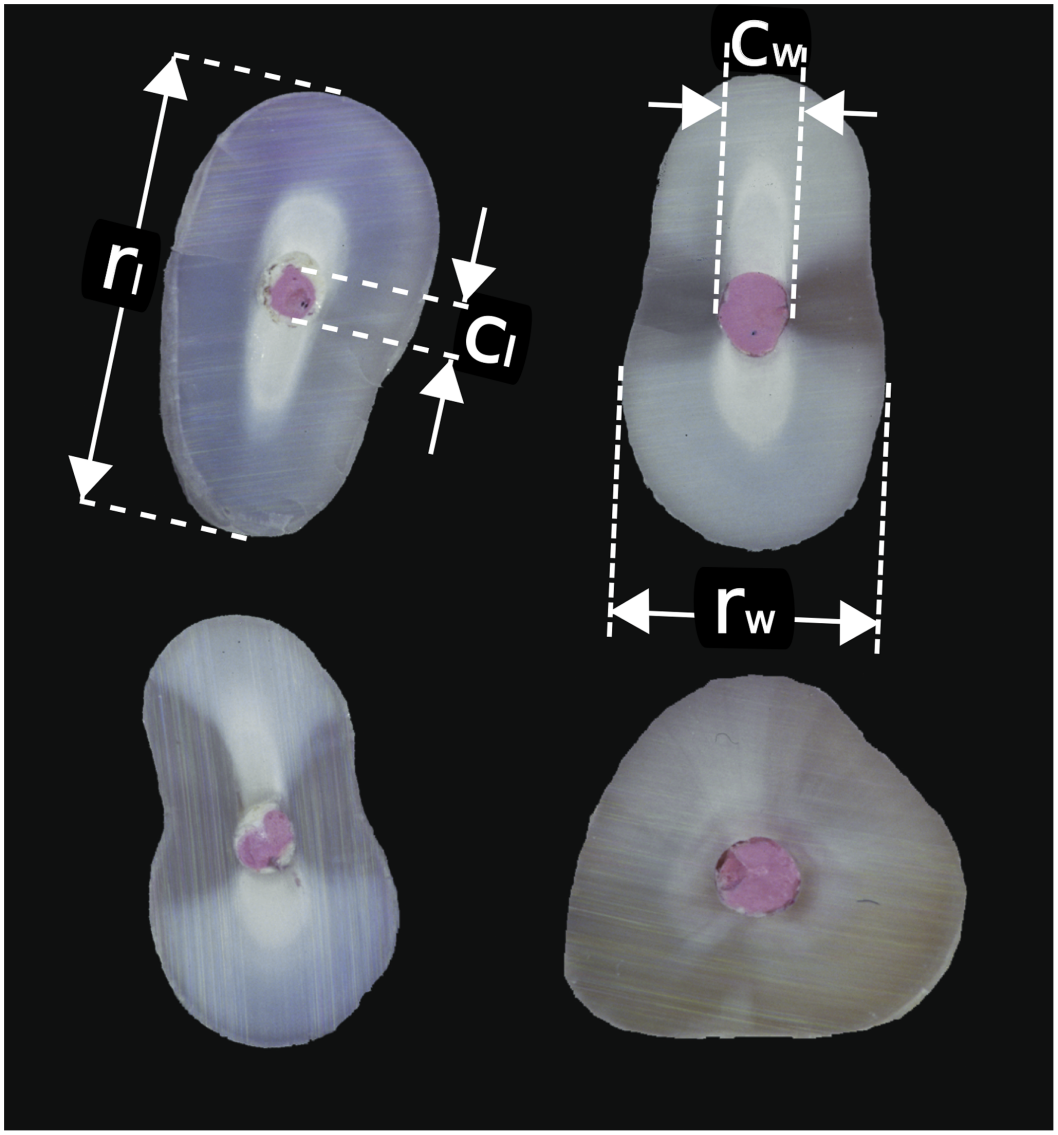
Cut faces of four root ends prior to the endodontists’ operating on them (root tips removed and pink gutta percha visible), showing some of the measures we took. For the upper left tooth we show the length of the long axis of the root acoss the root face (r_l_) and the length of the canal on the same axis (c_l_). For the upper right tooth, we show the width of the short axis of the root acoss the root face (r_w_), and the width of the canal on the same axis (c_w_). We repeated all measures after the teeth had been operated on by the endodontists, except that the central lengths and widths across the root face were of the cavities.

We photographed all the roots in a jig using standardized flash illumination at 3× magnification with 100 ASA colour transparency film. We digitized these images (Nikon Coolscan 5000 scanner, Nikon Corp, Tokyo, Japan) and measured them on a desktop computer using the Image J program (National Institutes of Health, Bethesda, MD, USA). Because the cross section of a root is not exactly circular, as in the classic Delboeuf illusion, but approximately oval, we defined two axes for our measurements: a long axis and a short axis. We refer to these below as lengths and widths respectively. We used the same axes for the canals and cavities. Examples of the measures we took at this stage are given in Figure 8.

After the endodontists had prepared the root-end cavities and returned the teeth we filled the root-ends with MTA (ProRoot MTA, Dentsply Tulsa Dental, Johnson City, TN, USA) using an MTA carrier, straight EPILA condensers and VA10 flat plastic instruments (G Hartzell and Son, Concord, CA, USA). This popular sealing material for surgical dental procedures also provided good photographic contrast in the absence of gutta percha. We then photographed and digitized the root ends under the same standardized conditions. These images were arranged in a random order and one of us made the same measurements, except that now he measured the cavities’ properties instead of the canals’.

To assess the reliability of the measures we regressed the measures of the roots after the teeth were worked on by three of the endodontists with the same measures before the teeth had been worked on by those endodontists. Because these are the same measures of the same teeth, if the measurement were perfectly reliable the slopes and intercepts of measures should all be 1 and 0 respectively. Slopes and intercepts were within 90% confidence intervals of 1 and 0 respectively.

### Statistical Analysis

We calculated linear regressions via least-squares. We tested slopes for significance with *F* tests against a null hypothesis of zero. We tested means for differences from zero with *t-*tests.

## Results and Discussion

### Overall Results

**Figure 9:**
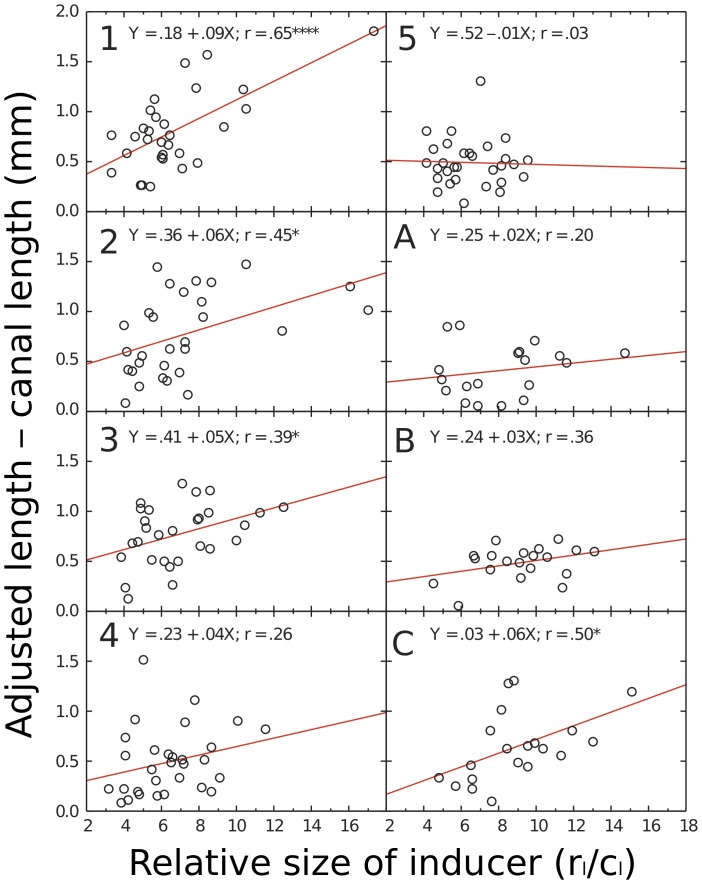
Relation between the potency of the Delboeuf illusion (relative inducer size) in teeth supplied to each endodontist to how much he or she cut into the tooth along the long axis of the root (adjusted length – canal length). Each graph shows the regression equation, the correlation coefficient for the relationship, and whether the relationship is statistically significant (*p<.05; ****p<.0001). In general, endodontists increased the length of cavities more in teeth showing a strong Delboeuf illusion than in teeth showing a weak Delboeuf illusion.

In Figure 9, we show scattergrams of how much the endodontists increased the lengths of the cavities (c_l_) over those of the canals plotted against the relative size of the inducer: the ratio of the long axis of the root face to the length of the canal on that axis (r_l_/c_l_). Each point on a scattergram represents one tooth. If endodontists are affected by the repulsion range of the Delboeuf illusion we should be able to fit a positively sloping regression line in each scattergram. (It is impossible for the endodontists to show that they were affected by the assimilation range of the illusion with teeth, unlike in Figure 2 from a computer display, because they could not make a hole smaller than it already was.) All but one endodontist (5) show such positively sloping regression lines; that endodontist fortuitously received teeth that did not yield a potent Delboeuf illusion of repulsion (see analyses of individual differences below). The mean slope is 0.04 (SD = 0.03); this is significantly greater than zero, *t*(7) = 4.10, *p*<.01, *d* = 1.41. The average variance of increase in long-axis size explained by the regression on the relative size of the inducer is 16%. These results suggest that endodontists are affected by the Delboeuf illusion when making cavities in teeth.

**Figure 10:**
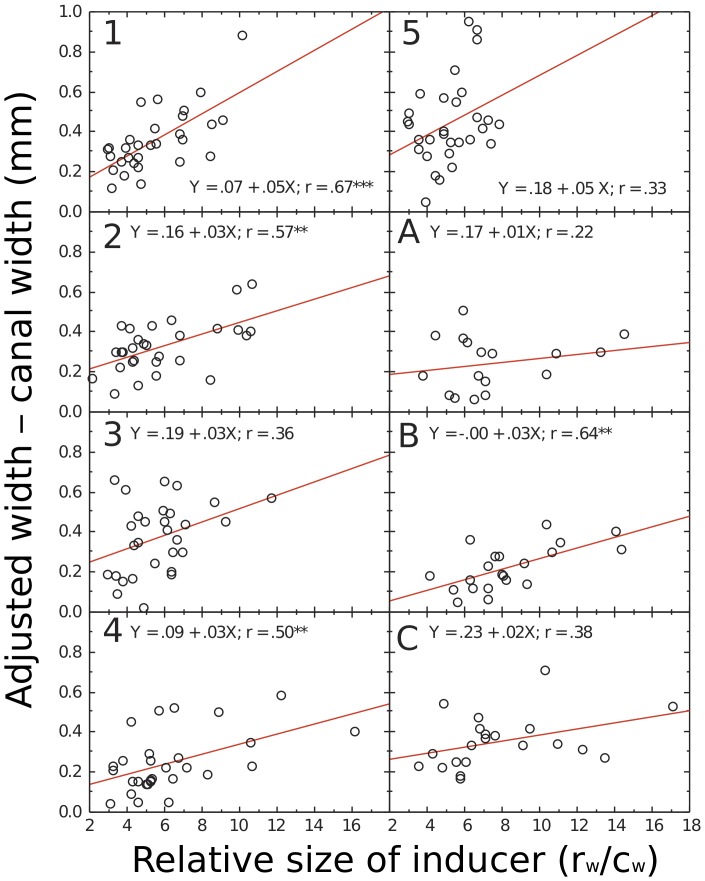
Relation between the potency of the Delboeuf illusion (relative inducer size) in teeth supplied to each endodontist to how much he or she cut into the tooth along the short axis of the root (adjusted width – canal width). Each graph shows the regression equation, the correlation coefficient for the relationship, and whether the relationship is statistically significant (*p<.05; ****p<.0001). In general, endodontists increased the width of cavities more in teeth showing a strong Delboeuf illusion than in teeth showing a weak Delboeuf illusion.

The same significant relationship between the increase in the width of the cavity (c_w_) and the relative size of the inducer exists across the short axis of the root face. We show these regression lines in Figure 10. All slopes are positive; the mean is 0.031 (0.14), significantly greater than zero, *t*(7) = 5.66, *p*<.001, *d* = 2.30. The average variance of increase in short-axis size explained by the regression on the relative size of the inducer is 23%. The mean of the short-axis slopes is not significantly less than that of the long-axis slopes, *F*(1, 7) = 1.49, *p*>.05. These results also suggest that endodontists are affected by the Delboeuf illusion when making cavities in teeth.

**Figure 11:**
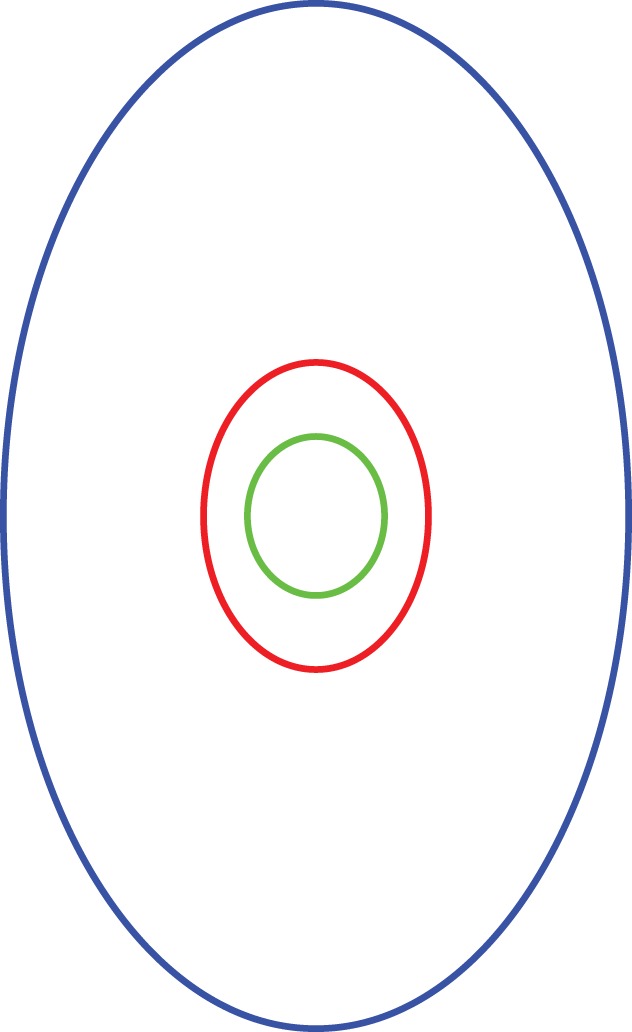
Scale drawing of the average shape of the roots across the root face (in blue), the average shape of the cavities endodontists made (in red), and the average shape of the canals of the teeth (in green). The canals were fatter ellipses than the cavities. The cavities resembled the shape of the roots more than of the canals.

Another indication that endodontists were affected by the visual context of the root face lies in the average dimensions of the cavities they cut. The average shape of the root faces was a thin ellipse (0.62 width-to-length ratio). The average shape of the root canals was a fat ellipse (0.74 ratio). Endodontists made their cavities have an average shape of a thin ellipse (0.66 ratio)–more like that of the root faces, a significant change, *F*(1, 120) = 31.13, *p*<.0001. This is illustrated in Figure 11.

### Individual Differences

To assess the individual differences in the slopes of the functions shown in Figures 9 and 10, we correlated them with various characteristics of the stimuli and of the endodontists, such as age and year of experience as an endodontist. (We could not include the slope of the function from the computer test of the illusion, shown in Figure 2, because only three of the endodontists did this test.) Long-axis slopes were positively correlated with the ranges of relative inducer sizes in the teeth, *r*(7) = .88, *p* = .0025. We propose that endodontists were affected by the illusion only when there was a reasonable range of the illusion among the teeth they received. Long-axis slopes were also positively correlated with years of experience as an endodontist, *r*(7) = .84, *p* = .0067, and with age, *r*(7) = .79, *p* = .0175: the more experienced, older ones were more affected by the illusion than the less experienced, younger ones. It is possible that this reflects increasing susceptibility to visual illusions with age [36], [37]. It is also possible that older endodontists are less familiar with today’s concepts of minimal intervention dentistry [38].

Because experience and age were also positively correlated, *r*(7) = .85, *p* = .0045, and because these variables also, because of sampling error, showed some correlation with the ranges of relative inducer sizes, *r*s(7) = .60 and.61, *p*s = .1182 and.1124, we conducted two stepwise multiple regressions on the long-axis slopes. Both used the range of relative inducer sizes as a predictor; one also used experience, the other age. In both analyses, the range of relative inducer sizes was the only significant predictor with an *F* to enter of 4.000 and an *F* to remove of 3.996. We conclude that the major influence on the slopes of the functions for the endodontists is the range of relative inducer sizes, arising simply from sampling error. We cannot rule out that age and experience also contribute to endodontists’ making longer cavities in teeth showing a potent value of the illusion ratio, but any such effect is minor.

Surprisingly, short-axis slopes were negatively correlated with the ranges of relative inducer sizes in the teeth, *r*(7) = –.72, *p* = .044. There were also no significant correlations between short-axis slopes and years of experience as an endodontist, *r*(7) = .21, *p* = .6274, and age, *r*(7) = .26, *p* = .5526. These differences from the pattern of results for long axis slopes seems to be due mainly to the influence of endodontist 5 whom we propose was doing something different from the other endodontists. When we removed endodontist 5’s data, the surprising correlation became non-significant, *r*(6) = –.61, *p* = .1540, and the other correlations became significant, *r*(6) = .79 *p* = .0335 for experience and *r*(6) = .95, *p* = .0002 for age. Endodontist 5 cut the most circular cavities of all, and was affected only by the range of relative inducer sizes only for the short axis, *r* = .33. We prefer to think that the same perceptual operations are at play for the short axes as for the long axes, although we are not aware of any research on oval Delboeuf figures to guide us here. Yet with endodontist 5’s data included, there is no significant correlation between short axis slopes and long axis slopes, *r*(7) = .06, *p* = .891. However, with endodontist 5’s data excluded there is a significant correlation between short axis slopes and long axis slopes, *r*(6) = .77, *p* = .040, consistent with our expectation. We are aware that the number of participants we have is quite small, but have some confidence in our belief that there are general operations because in this case reducing the number yielded significant results. We concede however, that more research needs to be done to clarify these individual differences.

## General Discussion

We have shown that endodontists cut larger cavities in teeth that exhibit a strong Delboeuf illusion of repulsion than they cut in teeth that exhibit a weak Delboeuf illusion. Across the long axes of the teeth, on average, the endodontists increased the size of the canals by about 0.59 mm when the relative size of the inducer had a neutral value of 7 and by about 0.97 mm, 1.65 times bigger, when the relative size of the inducer had a potent value of 16. Across the short axes of the teeth, on average, the endodontists increased the size of the canals by about 0.36 mm when the relative size of the inducer had a neutral value of 7 and by about 0.64 mm, 1.79 times bigger, when the relative size of the inducer had a potent value of 16. About 50% of the teeth we used had values of the relative size of the inducer larger than the neutral value of 7 suggesting that the illusion could be affecting endodontists’ performance quite commonly.

Before we can accept that the Delboeuf illusion caused the increased cavity sizes, we need to consider alternative explanations. Knowing only the regressions shown in Figures 9 and 10, one could argue that endodontists enlarged small cavities by a fixed amount to allow the passage of instruments. But if this were so, there would be no need to make the cavities have a thinner shape than the holes. This is plausible only if the endodontists were affected by the context of the holes.

One could also argue that the endodontists had thought (erroneously) that there would be more potentially infected material present in the root ends of large teeth than in small teeth, and that a larger cavity was required to eliminate this (and that moreover this was safer to do in larger teeth). We can reject this for three reasons:

1. There were no significant correlations between the increased size of cavities and the size of teeth. The significant correlations emerged only when the size of the canal was expressed as a ratio with the size of the root face.
2. There would be no need to enlarge the long axis of their cavities more than the short axis.
3. The endodontists were specialists with extensive knowledge and experience, so would not have made such an error.

One could also argue that there was a problem with external validity. The endodontists were operating on isolated teeth, clearly not in patients’ mouths. Perhaps they were more casual with these teeth, allowing themselves to be affected by the illusion when they would not be so affected by teeth in the patient’s mouth. But this seems unlikely because all the endodontists were highly experienced; in the same period as each one was operating on our experimental teeth, he or she would have performed comparable procedures for patients. We have no reason to doubt that the endodontists would have done anything but the same careful task on the experimental teeth, especially because they knew that we intended to scrutinize their work in some way.

If it can be accepted that the Delboeuf illusion led the endodontists to cut larger cavities than necessary, then we have given an example of when visual perception affects treatment. In root-end surgery, there are at least two sequelae of removing more healthy tooth than necessary: cracking of the root end and perforation of the root end [33] because this region has less tooth tissue than text books suggest [39]. If endodontists cut larger cavities than needed because of the Delboeuf illusion, these adverse events will be more likely.

If it is accepted that visual illusions can influence treatment in endodontics, then it is possible that they influence treatment (and diagnosis) in other dental procedures (such as drilling out caries in the crowns of teeth). In fact dentists have long been aware of the importance of visual perception and illusions for a cosmetically acceptable result of dental work [17], [40], [41], [42], [43], however we believe our study is the first to show that an illusion affects treatment.

If it is accepted that visual illusions can influence treatment in dentistry, then it is possible that they influence treatment (and diagnosis) in other fields of health care. Again, other health-care practitioners are aware of the importance of illusions for producing a cosmetically pleasing result [22] and in diagnosis or treatment [16], [18], [19], [21], [44], [45]. We chose a model system that bore a close resemblance to the classic Delboeuf illusion, with rigid, near-circular shapes. We used a prospective approach. We note that similar displays are common in medicine and surgery (e.g., nerves, vessels, the cross sections of many bones, and some radiographic structures). In any event, the Delboeuf illusion is not confined to such shapes but represents a general effect of the size of a context.

The question then becomes how to prevent health practitioners from being influenced by visual illusions. One answer is in a practitioner’s simply being aware of the potential for visual illusions to affect treatment [46]. It has also been shown that taking an analytical attitude [47] moving the eyes over the object of interest [48], and receiving corrective feedback [49] can reduce, although not eliminate, the influence of visual illusions. A better answer might lie in using measurement, because measuring instruments are not subject to visual illusions.
